# Insights from the use of multipolar mapping during ablation of supraventricular and ventricular arrhythmias

**DOI:** 10.1016/j.hroo.2025.01.005

**Published:** 2025-01-19

**Authors:** Eric D. Braunstein, James K. Gabriels, Ashkan Ehdaie, Jonathan Yarnitsky, Hailei Liu, Archana Ramireddy, Xunzhang Wang, Michael Shehata

**Affiliations:** 1 Department of Cardiology, Smidt Heart Institute, Cedars-Sinai Medical Center, Los Angeles, California; 2 Cardiovascular Institute, North Shore University Hospital, Manhasset, New York; 3 Department of Research and Development, Biosense Webster, Irvine, California

**Keywords:** Mapping, Electrogram, Electroanatomic mapping, Multipolar, Bipolar, Unipolar

## Abstract

**Background:**

Multipolar mapping, a novel method of electrogram acquisition and annotation distinct from unipolar and bipolar acquisitions, provides orientation-independent near-field signal acquisition with enhanced spatial precision. Multipolar mapping use in humans has not been described.

**Objective:**

This study aimed to present findings on the use of multipolar mapping in a variety of different arrhythmia types and mechanisms.

**Methods:**

We performed consecutive ablation procedures using a high-density multipolar-capable mapping catheter (OPTRELL, Biosense Webster, Irvine, CA). Parallel mapping using multipolar signal acquisition and conventional bipolar signal acquisition was performed during ablation of various supraventricular and ventricular arrhythmias. Multipolar and bipolar voltage and local activation time maps were compared. A comparison was also made between multipolar, bipolar, and unipolar electrograms in areas of interest.

**Results:**

During ablation to treat atrial fibrillation, atrial tachycardia, accessory pathway, premature ventricular contractions, and ventricular tachycardia, we observed 4 advantages of multipolar mapping compared with traditional bipolar mapping: (1) an improved ability to remove far-field signals while preserving the local waveform, (2) wavefront direction independence, (3) a more accurate representation of voltage, and (4) improved ability to identify the origin of focal arrhythmias.

**Conclusion:**

In this small observational study of multipolar mapping during ablation of various “real-world” arrhythmias, several advantages of multipolar mapping are demonstrated. Larger studies including evaluation of patient outcomes will help further define the benefit of this novel mapping strategy.

**Key Findings**

▪ Multipolar mapping is a novel method of electrogram acquisition and annotation distinct from unipolar and bipolar acquisitions, which provides orientation-independent near-field signal acquisition with enhanced spatial precision.
▪ Multipolar mapping displays an improved ability to remove far-field signals while preserving the local waveform.
▪ Multipolar electrograms are wavefront direction independent.
▪ Multipolar maps may give a more accurate representation of voltage.
▪ Multipolar mapping may improve the ability to identify the origin of focal arrhythmias.

## Introduction

Electrogram recording, which is the basis of invasive electrophysiology (EP), relies on the notion that measured extracellular potentials are related to intracellular voltage changes during myocardial cell depolarization.^1^^,^^2^ Electrogram acquisition has primarily been either unipolar (a single intracardiac pole with a distant extracardiac reference electrode) or bipolar (2 closely spaced intracardiac electrodes). Both acquisition types have disadvantages, including that unipolar electrograms are subject to far-field signals and noise and bipolar electrograms are orientation dependent relative to the wavefront of activation.^3^^,^^4^ Although bipolar electrogram acquisition is primarily used in clinical practice, unipolar acquisition is useful in several circumstances, including identification of the origin of focal arrhythmias,^5^ during mapping of accessory pathways,^6^ and identification of the local activation time (LAT) in multicomponent bipolar signals.^7^

Multipolar electrograms and multipolar mapping are a novel method of electrogram acquisition that are distinct from both unipolar and bipolar acquisitions. This method combines the benefits of both unipolar and bipolar electrograms. Using a fixed electrode array catheter (OPTRELL, Biosense Webster, Irvine, CA), a “near-field” unipolar electrogram is constructed. The combination of both bipolar and unipolar electrograms allows orientation-independent near-field signal acquisition with enhanced spatial precision.^8^

To date, no studies on the “real-world” use of multipolar electrograms during electroanatomic mapping in humans have been performed. In this study, we present our findings on the use of multipolar mapping in a variety of different arrhythmia types and mechanisms.

## Methods

Consecutive ablation procedures performed at a single institution, including treatment of various arrhythmia mechanisms and sources, were studied. Mapping was performed using the Biosense Webster OPTRELL catheter (36-pole, D/F curve), with CARTO 3 version 8.1 including the CARTO ELEVATE module. High-density “parallel” mapping was performed with both multipolar acquisition and annotation (multipolar peak −dV/dt) and conventional bipolar acquisition with “wavefront” annotation (unipolar peak −dV/dt). Cases with significant findings or differences between multipolar and bipolar acquisitions, defined by differences seen on either voltage or activation maps or differences between multipolar and bipolar electrograms, were identified. This study was approved by the Institutional Review Board at Cedars-Sinai Medical Center. Because of the retrospective nature of the study, patient consent was waived by the institutional review board. This study was conducted in accordance with the ethical principles outlined in the Declaration of Helsinki.

**Figure 1:**
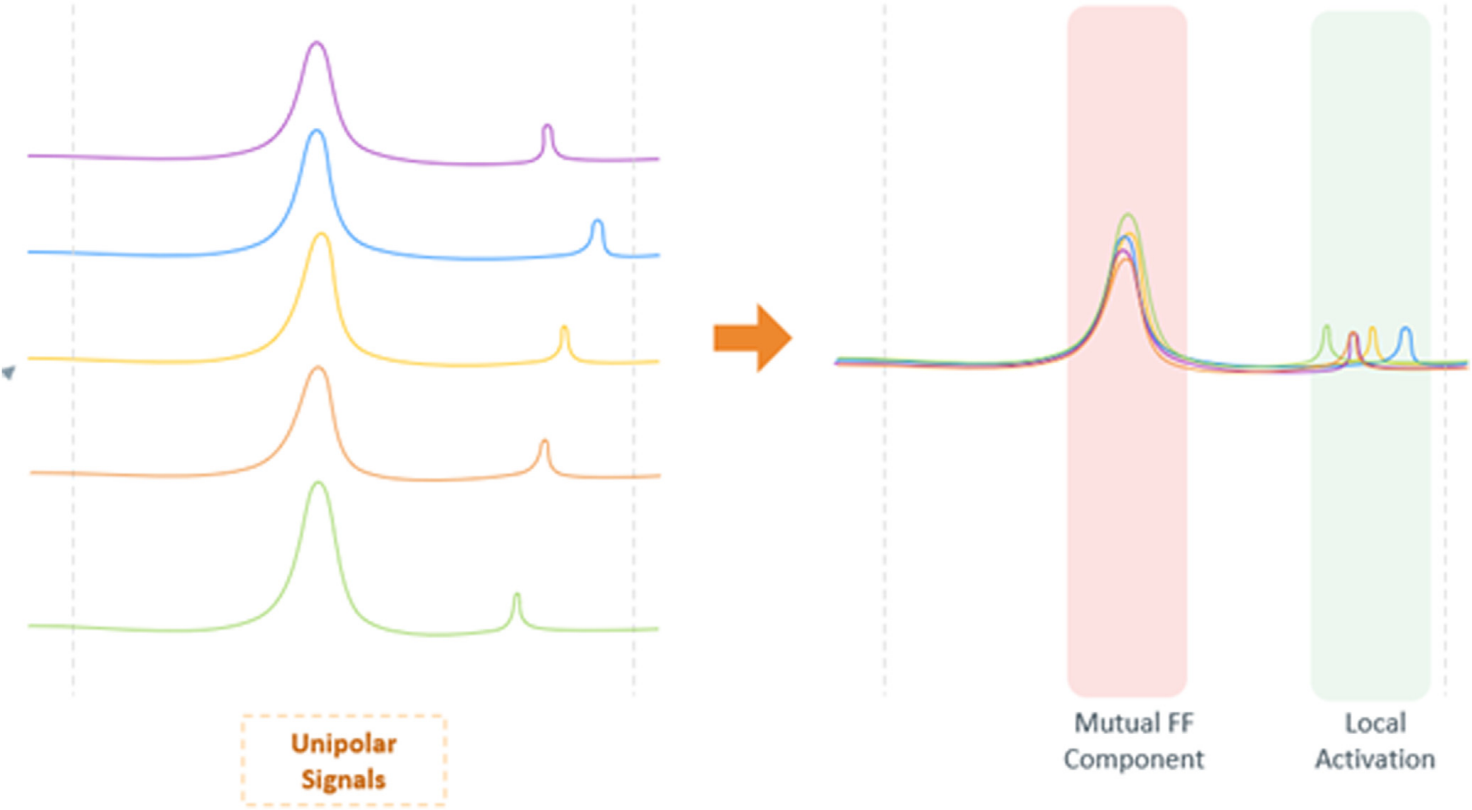
Schematic diagram of multipolar signal acquisition. The mutual far-field (FF) component is subtracted from each unipolar electrogram, resulting in an isolated “near-field” unipolar signal.

The multipolar algorithm introduces a novel and efficient approach to far-field reduction in unipolar signals while preserving true local activation. Far-field characteristics are location dependent, meaning that the far-field signal captured by an electrode would have characteristics similar to those of other electrodes within its immediate vicinity. The algorithm uses the unique multielectrode array catheter shape and spatial surrounding information to eliminate the common components of each unipolar signal with its neighboring electrodes by using principal component analysis. A mathematical multisignal decomposition method is used to separate the near-field and far-field contributions to the electrogram, such that the largest common signal component hidden within all electrograms is identified as the far-field contribution. Once identified, this largest common signal component is scaled to best fit the electrode signal being inspected and then subtracted from that electrode unipolar signal. What is left after the subtraction is a residual signal that estimates pure local information. As noted, multipolar signal activation annotation is based on the signal’s sharpest −dV/dT slope, representing the exact timing of wavefront conduction passing underneath the electrode (Figure 1).

Multipolar and bipolar LAT and voltage maps were analyzed for procedures involving several arrhythmia mechanisms and chambers of interest, along with relevant corresponding multipolar, bipolar, and unipolar electrograms collected by the mapping system. Annotation of all electrograms was performed automatically by the Carto system. Manual reannotation of electrograms was not performed.

## Results

Approximately 25 cases were performed during the study period where parallel multipolar and bipolar mapping was performed. Six cases of various arrhythmia mechanisms were selected where significant differences between multipolar and bipolar maps and/or electrograms were seen.

### Supraventricular arrhythmias

#### Paroxysmal atrial fibrillation

**Figure 2:**
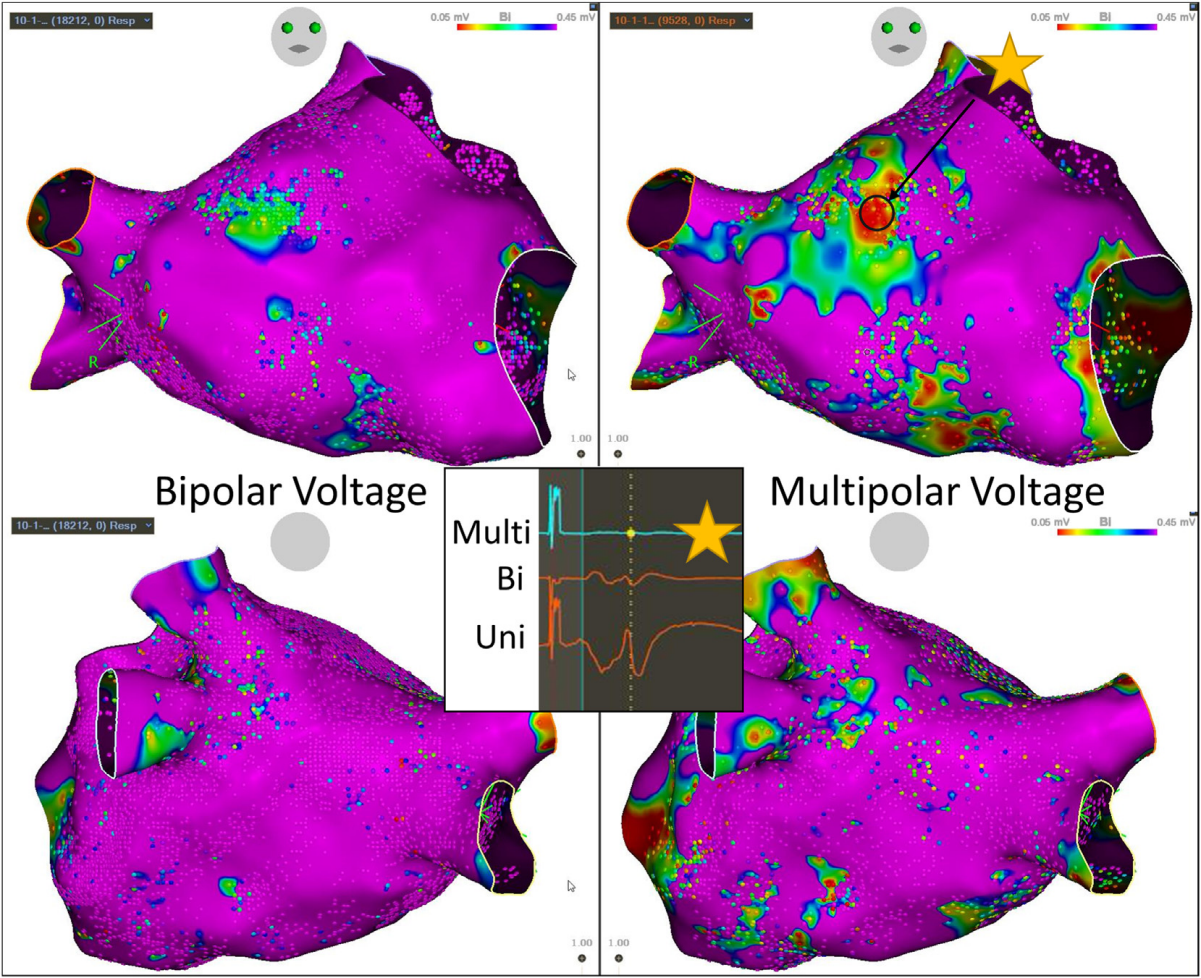
Bipolar and multipolar voltage maps during atrial paced rhythm from a patient with paroxysmal atrial fibrillation undergoing repeat ablation. The *inset* illustrates an area of low voltage that is accentuated on the multipolar map and electrogram.

During a repeat procedure for a patient with paroxysmal atrial fibrillation (AF), parallel mapping was performed using bipolar and multipolar electrograms in the left atrium. Both maps revealed reconnected pulmonary veins and scattered areas of abnormally low endocardial voltage. Regions of low voltage were better defined and accentuated on the multipolar voltage map (Figure 2). Analysis of electrograms in the areas of low voltage on the multipolar map demonstrated an improved elimination of far-field–appearing atrial electrograms when compared to the bipolar map (Figure 2, inset). Elimination of far-field electrograms likely accounted for the accentuation of the low-voltage areas.

#### Persistent AF

A 77-year-old man with a complex medical history including multivessel coronary artery disease with prior percutaneous and surgical coronary interventions, mitral valve repair, heart failure with a reduced ejection fraction of 39%, and persistent AF and atrial flutter presented for ablation. Complex ablation for AF and atrial flutter consisted of retrograde venous ethanol ablation of the vein of Marshall, pulmonary vein isolation, lateral mitral line, and posterior wall isolation with posterior rooflines and floor lines. Postablation mapping during distal coronary sinus pacing, focused on the lateral mitral isthmus, was performed with parallel mapping of bipolar and multipolar electrograms.

**Figure 3:**
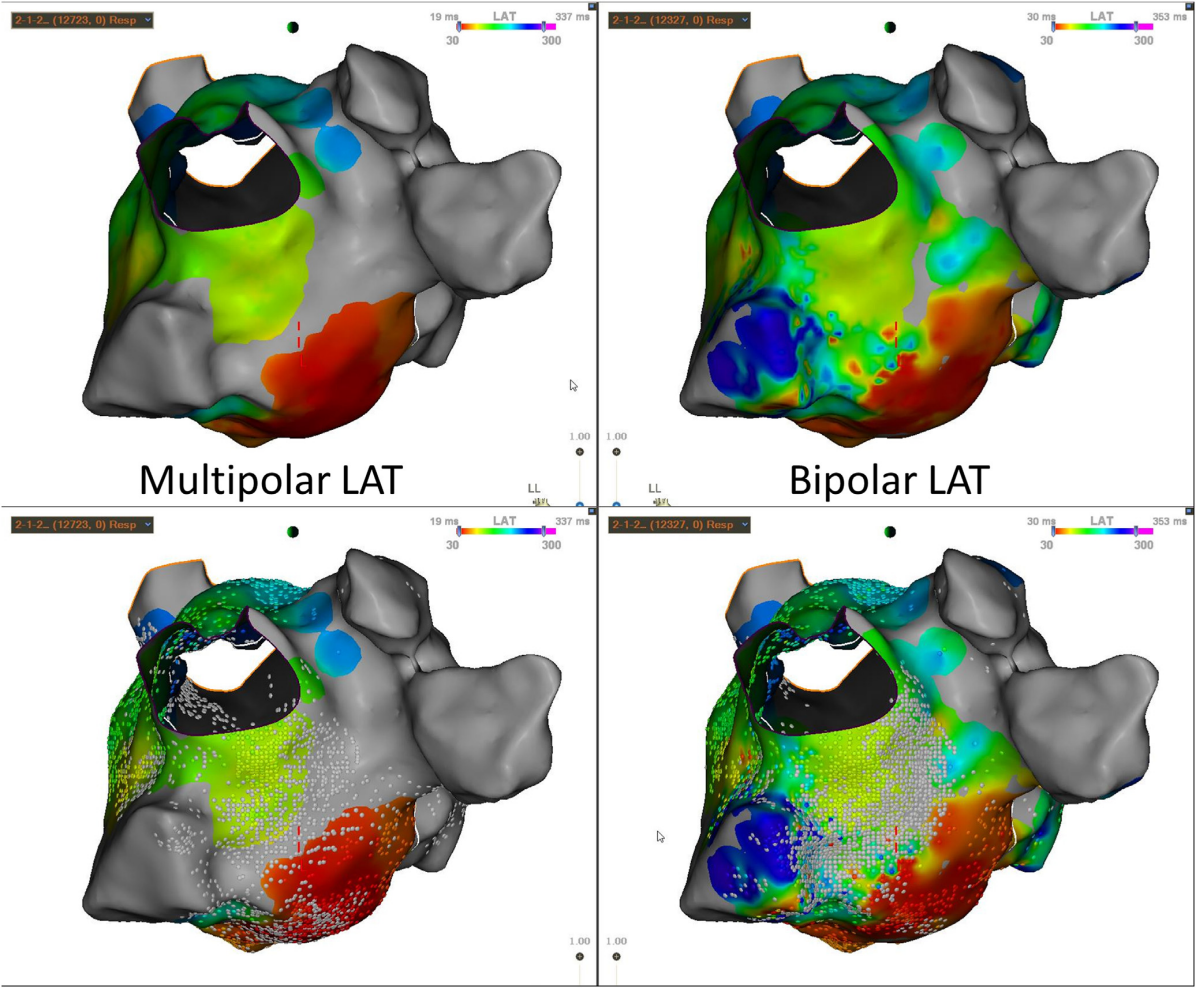
Multipolar and bipolar local activation time (LAT) maps after creation of a lateral mitral isthmus line during distal coronary sinus pacing. Maps without (*above*) and with (*below*) LAT points are shown.

Multipolar mapping revealed an improved ability to delineate the lateral mitral isthmus ablation line, with less annotation of far-field electrograms along the line (Figure 3), making it more evident that unidirectional block was present. Bidirectional block was also confirmed using differential pacing.

#### Complex atrial tachycardia

**Figure 4:**
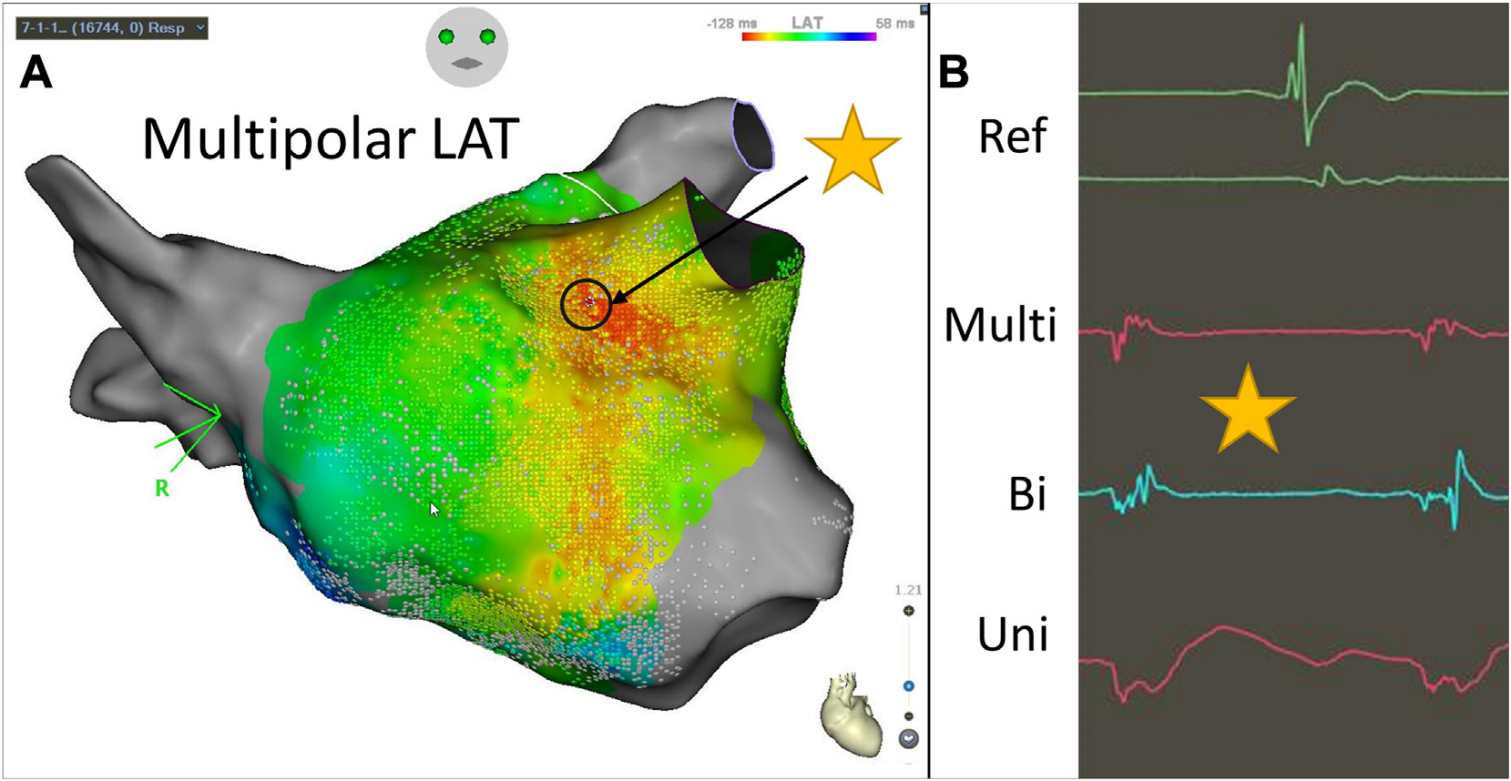
Multipolar local activation time (LAT) map and corresponding electrograms at the earliest activation site of a focal atrial tachycardia. The star represents where the displayed electrogram on the right correspond to anatomically on the electroanatomic map.

During persistent AF ablation using radiofrequency energy, after retrograde venous ethanol ablation of the vein of Marshall, pulmonary vein isolation, posterior wall isolation, lateral mitral line, and cavotricuspid isthmus ablation, programmed stimulation resulted in an induction of an atrial tachycardia. Entrainment suggested a non-reentrant mechanism on the basis of lack of progressive fusion, lack of a consistent postpacing interval, and instability in the tachycardia cycle length after pacing maneuvers. An activation map was created in the left atrium. The activation map was consistent with a focal atrial tachycardia arising from the medial base of the left atrial appendage (Figure 4A). At the site of earliest activation, a relatively narrow “QS” multipolar electrogram was seen. A “QS” unipolar electrogram was also noted, whereas the bipolar electrogram was biphasic and fractionated (Figure 4B).

#### Right lateral accessory pathway

**Figure 5:**
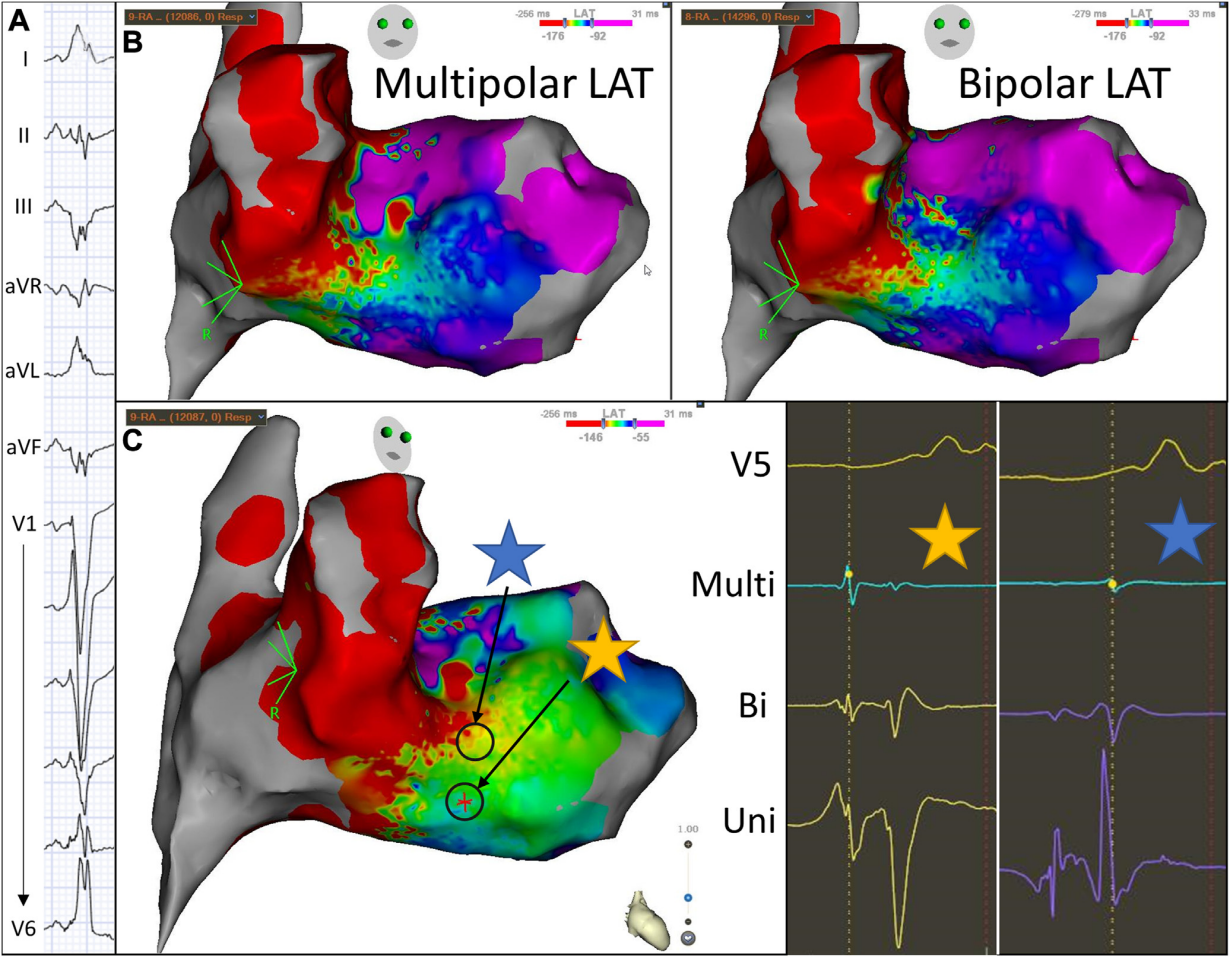
Electrocardiogram, local activation time (LAT) maps, and corresponding electrograms from a patient with preexcitation. See text for details. The star represents where the displayed electrogram on the right correspond to anatomically on the electroanatomic map.

A 43-year-old woman with manifest preexcitation on the electrocardiogram presented for EP study and ablation. The electrocardiogram suggested a right lateral accessory pathway (Figure 5A). The baseline EP study demonstrated an accessory pathway with anterograde-only conduction. Open window parallel mapping was performed along the lateral tricuspid annulus during sinus rhythm, in which a wide mapping window was set to include atrial and ventricular timed signals. Multipolar and bipolar LAT maps were overall similar (Figure 5B). Analysis of multipolar electrograms from points along the tricuspid annulus showed better delineation of the annular plane compared with analysis of bipolar electrograms, with improved ability to eliminate far-field atrial signals (Figure 5C, blue star) and ventricular signals (Figure 5C, yellow star), enhancing the ability of the open window map to correctly identify the annulus. After ablation along the lateral tricuspid annulus, pathway conduction was eliminated.

### Ventricular arrhythmias

#### Premature ventricular contractions

**Figure 6:**
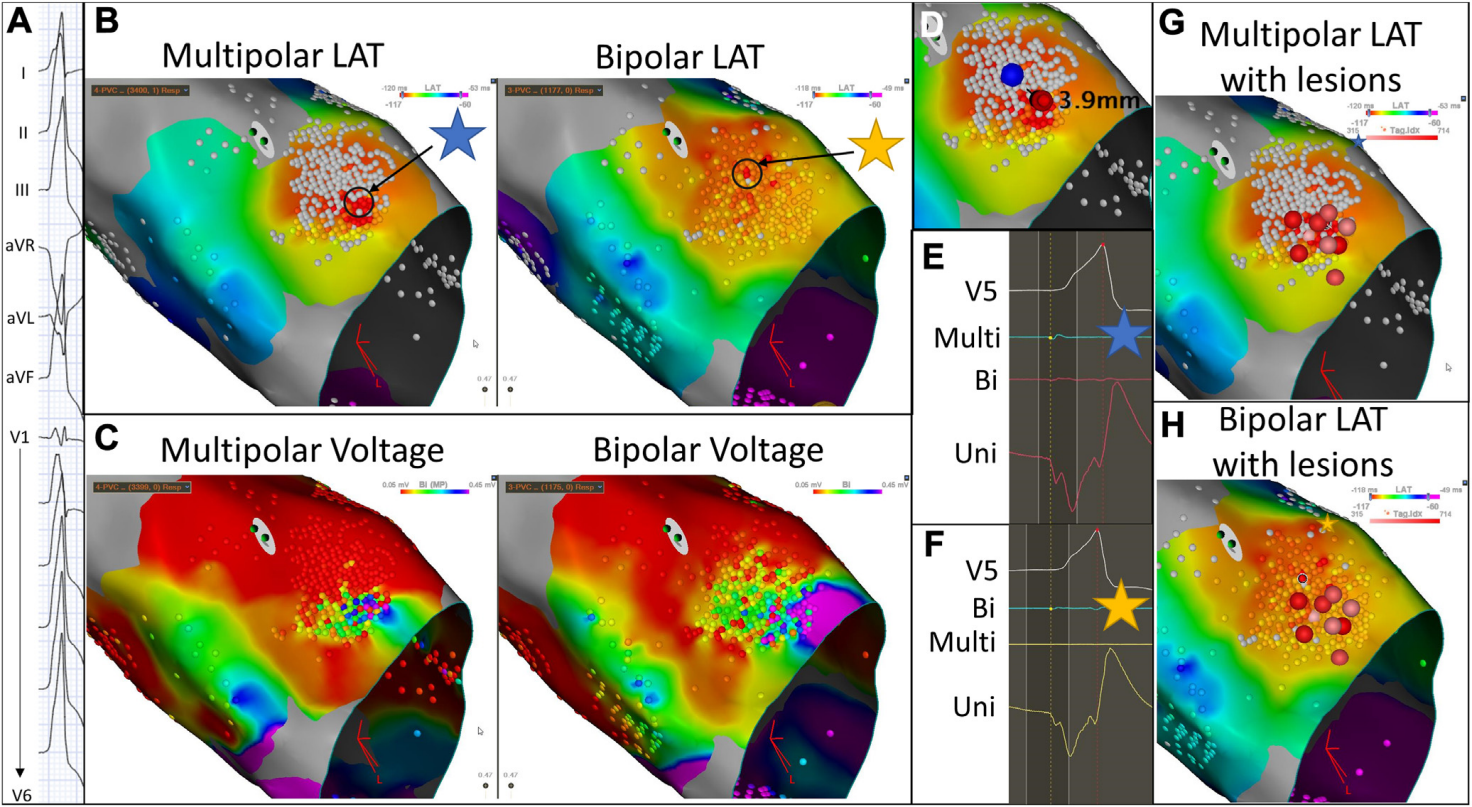
Multipolar and bipolar local activation time (LAT) and voltage maps and corresponding electrograms. See text for details. The star represents where the displayed electrogram on the right correspond to anatomically on the electroanatomic map.

During ablation to treat symptomatic monomorphic premature ventricular contractions (PVCs) with a right bundle, left inferior axis, positive precordial concordance, and notching in lead V_1_ (Figure 6A), the left ventricular outflow tract was mapped via a retrograde aortic approach (Figures 6B and 6C). Parallel mapping was performed using bipolar and multipolar electrograms. The earliest ventricular activation was noted in the left coronary cusp near the right-left coronary cusp commissure. At this location, the local electrogram preceded the QRS onset by 35 ms when the clinical PVC occurred (Figures 6E and 6F). The LAT during PVCs in the right ventricular outflow tract opposite this area occurred at the onset of the QRS complex.

The earliest LAT activation area with multipolar mapping was more focused than that with bipolar mapping (Figure 6B). Multipolar voltage mapping showed a more linear demarcation at the myocardial-arterial interface at the top of the left ventricular outflow tract than did bipolar mapping (Figure 6C). The earliest point on multipolar and bipolar mapping was separated by 3.9 mm (Figure 6D).

Electrogram analysis at sites of earliest activation on multipolar and bipolar mapping was performed. At the site of earliest multipolar activation, multipolar and unipolar “QS” complexes were seen and corresponding small bipolar electrograms were seen (Figure 6E). At the site of earliest bipolar activation, a very small bipolar electrogram was seen with a corresponding “QS” unipolar electrogram, and no corresponding multipolar electrogram was seen there (Figure 6F). Ablation was performed at the site of earliest multipolar activation with immediate PVC suppression, and a small cluster of additional ablation was performed for consolidation (Figure 6G). No ablation was performed immediately at the site of earliest bipolar activation (Figure 6H). The patient remained free of PVCs during follow-up.

#### Ventricular tachycardia

**Figure 7:**
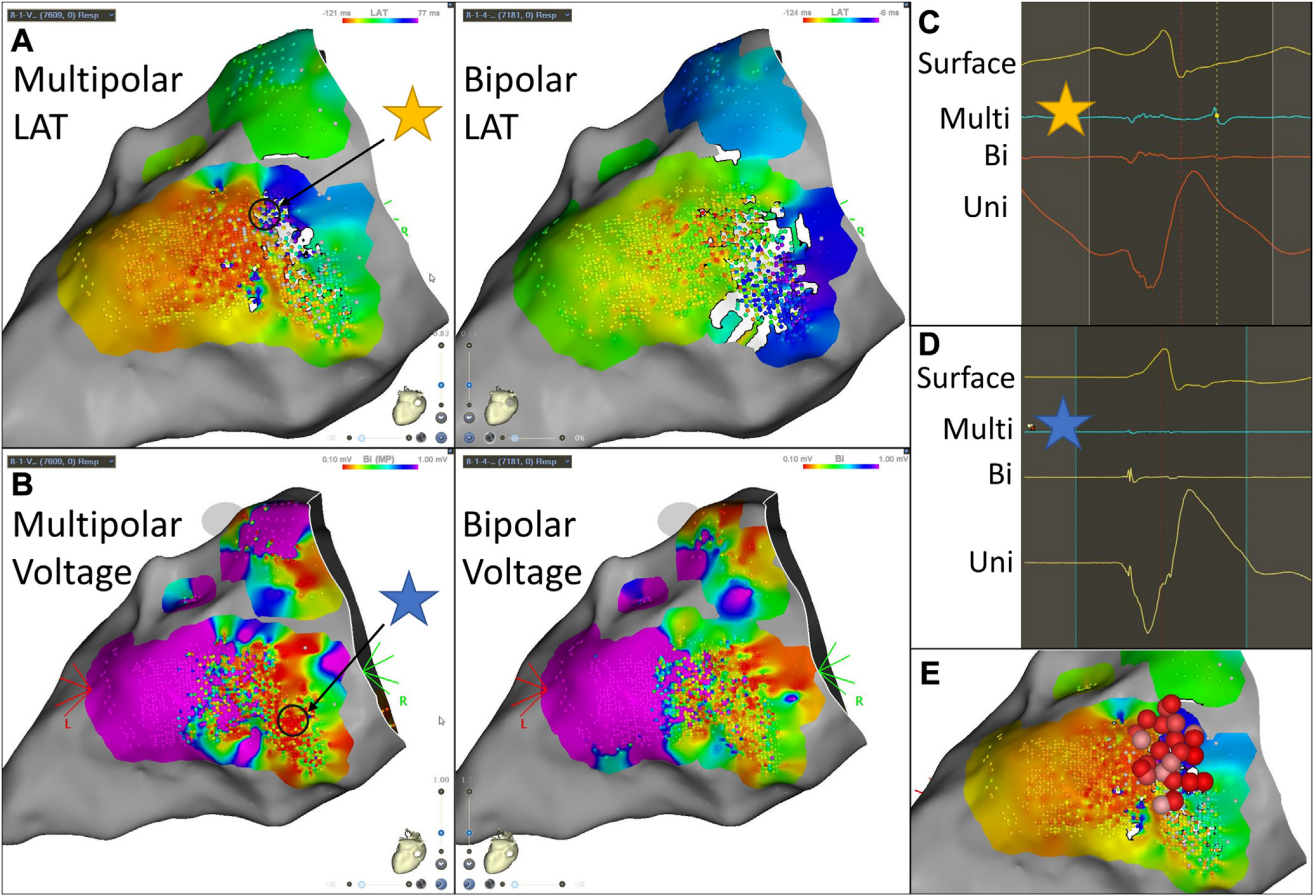
Local activation time (LAT) and voltage maps during ischemic ventricular tachycardia ablation. See text for details. The star represents where the displayed electrogram on the right correspond to anatomically on the electroanatomic map.

During ischemic ventricular tachycardia (VT) ablation, a VT with a right bundle branch, right superior axis, and V_5_ reverse transition was induced. Endocardial left ventricular mapping was performed during sinus rhythm and during VT. Compared with bipolar mapping, multipolar mapping during VT showed a clearer isthmus and VT circuit in the basal lateral left ventricle (Figure 7A). At a region where mid-diastolic multipolar electrograms were noted, the corresponding bipolar electrograms at the same location were much less apparent, and these mid-diastolic signals were not annotated on the bipolar map, making identification of the VT isthmus much more difficult (Figure 7C). Substrate mapping during paced rhythm showed a more discrete area of low voltage in the basal inferolateral left ventricle (Figure 7B). Analysis of very low voltage multipolar electrograms showed a larger corresponding unipolar electrogram (Figure 7D).

A moderate-sized cluster of radiofrequency ablation was performed in the area of the isthmus seen on the multipolar map (Figure 7E). This lesion set was extended inferiorly along the inferolateral border zone between normal and low endocardial voltage. The clinical VT was noninducible after ablation. The patient remained without further VT during follow-up.

## Discussion

Multipolar acquisitions leverage the synergy between bipolar and unipolar electrograms, representing an advancement in the fundamentals of invasive EP. Several main advantages were identified when using multipolar electrograms: (1) ability to remove far-field signals while preserving the local waveform, (2) wavefront direction independence, (3) a more accurate representation of voltage, and (4) an improved ability to identify the origin of focal arrhythmias.

Multipolar displays refined the LAT maps while improving precision and accuracy in the cases shown. During ischemic VT ablation, multipolar maps showed better preservation of the local waveform than did bipolar maps. A larger portion of the VT cycle length could be mapped, and the central isthmus was more readily apparent without manual reannotation of the electrogram. During open window mapping in a patient with manifest preexcitation, multipolar acquisitions showed an improved ability to map both chambers simultaneously and a greater ability to remove far-field electrograms along the annulus. Multipolar acquisitions showed clearer delineation of a line of block through far-field subtraction after ablation of the mitral isthmus during complex AF ablation.

Unlike bipolar electrograms that are fundamentally orientation dependent, multipolar electrograms are orientation independent and may improve signal quality and local activation precision with various catheter orientations. This, along with improved ability to remove far-field signals, likely played a role in improved identification of the VT circuit in reentrant VT.

Delineation of abnormally low voltage may be more accurate with multipolar mapping. Smaller but more discrete areas of low voltage were seen on multipolar maps than on bipolar maps. This is of particular importance in small areas of myopathic tissue, such as in patients with paroxysmal AF, and in focal arrhythmias arising adjacent to myocardial-nonmyocardial interfaces (ie, semilunar valves), such as in the ventricular outflow tracts.

The site of origin of focal arrhythmias has traditionally been identified in 2 ways: by identifying the earliest activation point with bipolar electrograms and by verifying that this site corresponds to a “QS” morphology on the unipolar electrogram. This finding suggests that activation occurs only in a direction away from this site of presumed origin.^9^ Similar to a unipolar electrogram, the multipolar electrogram is focal and orientation independent and therefore should display a similar “QS” electrogram as the site of origin of focal arrhythmias. We found this to be the case during mapping of both focal atrial and ventricular arrhythmias, with improved ability to identify the origin when compared with using bipolar electrograms. As expected, the multipolar “QS” pattern corresponded with a unipolar “QS” pattern, but this was not reflected in bipolar electrograms.

### Limitations

Potential limitations of multipolar mapping include the potential to eliminate “far-field” electrograms when these electrograms may represent the electrograms of interest. For example, when a mid-myocardial or epicardial substrate is mapped from the endocardial surface, the substrate of interest may be more well represented by the “far-field” electrogram, which will be removed with multipolar mapping.^10^ The multipolar mapping technique is also dependent on catheter-tissue contact between the multielectrode catheter and the tissue of interest, so proper catheter positioning while mapping is important, and further research will be needed to assess the performance of multipolar mapping of intracavitary structures such as the papillary muscles.

## Conclusion

In this small observational study of multipolar mapping during various real-world arrhythmias, we showed several advantages compared with bipolar mapping. Comprehensive studies in a larger population assessing outcomes may help to further illustrate the advantages of multipolar mapping.
